# Mechanotransduction of autotransplants: remodeling potential of heart valves from autologous pericardial tissue

**DOI:** 10.3389/fbioe.2025.1680107

**Published:** 2025-10-02

**Authors:** Marvin Steitz, Mahamuda Badhon Khan, Alexander Breitenstein-Attach, Boris Warnack, Frank Edelmann, Felix Berger, Boris Schmitt

**Affiliations:** ^1^ Department of Congenital Heart Disease – Pediatric Cardiology, Deutsches Herzzentrum der Charité, Berlin, Germany; ^2^ Corporate Member of Freie Universität Berlin and Humboldt-Universität zu Berlin, Charité – Universitätsmedizin Berlin, Berlin, Germany; ^3^ German Centre for Cardiovascular Research, Berlin, Germany; ^4^ Warnack MedConsult, Basel, Switzerland

**Keywords:** biomaterials, heart valve prosthesis, autograft, pericardium, cardiology, surgery, mechanotransduction

## Abstract

Current commercial heart valve prostheses are non-living structures, either derived from artificial materials (mechanical valves) or foreign biological materials (xeno- or homo-graft). Since the use of viable tissue with native-like properties is essential for a heart valve with self-regulation properties, autologous collagen-based tissue can be considered a promising alternative material. While the extracellular matrix of pericardial tissue offers a solid foundation, it is the interstitial cells that play a crucial role in ensuring long-term durability. This review explores the mechanotransduction capabilities of autologous tissue as a replacement material for living heart valves with regenerative potential.

## Introduction

Heart valves constructed from autologous native pericardial tissue (NPT) represent a promising alternative to commercial heart valves, whether mechanical or biological (homografts or xenografts). The Ozaki procedure has already demonstrated favorable long-term outcomes, with follow-up data extending up to 15 years (Fujikawa et al., 2025; Lansakara et al., 2023). Another prosthesis made from autologous NPT is the minimally invasive implantable GrOwnValve, which has also shown potential for promising long-term outcomes (Ayyash et al., 2025).

These heart valve prostheses may either remain viable (Gardin et al., 2021) due to intraoperative manufacturing or become recellularized, as the collagen-based tissue offers a highly cell-adhesive surface (Schornik et al., 2012). Consequently, autologous pericardial heart valves could retain or regain their self-regulatory properties (Zhai et al., 2006), allowing them to remodel into a native-like valve structure. This process, in which tissue adapts to its surrounding environment in response to mechanical forces, is known as mechanotransduction (Tietze et al., 2020).

### Comparison of clinical application scenarios

The heart valve replacement store offers a wide variety of biological prostheses of xenogenic origin (Yepez and Ríos, 2021), whose durability and functionality have evolved significantly over the past 15 years, resulting in a substantial increase in their application compared to mechanical prostheses (Rodriguez-Gabella et al., 2017). However, this trend comes at the cost of potential structural valve degeneration (SVD) (Bezuidenhout et al., 2009), which was described as the main failure reason of transcatheter heart valves elsewhere (Trimaille et al., 2025). Since bioprosthetic valves are non-viable substitutes (Sodian, 2004), incapable of regeneration or remodeling (Rodriguez-Gabella et al., 2017), their degeneration is inevitable, typically beginning 7–8 years after implantation (Kostyunin et al., 2020). It was reported that the absence of remodeling already becomes apparent as early as 6 months post-implantation, marked by irreversible damage to collagen molecules (Kostyunin et al., 2020). In pediatric patients, in which bioprosthetic valves are also the preferred choice (Ayyash et al., 2025), they face the additional limitation of being unable to adapt to the children’s somatic growth (Rodriguez-Gabella et al., 2017), leading to re-operations due to failure and outgrowth of the prostheses (Fioretta et al., 2021). These limitations could be overcome by utilizing autologous tissue, which has the capacity to be constructed into a regenerative prosthesis as described elsewhere (Straka et al., 2017).

The data available on the durability of transcatheter heart valves is very limited. However, transcatheter aortic valve functionality was reported to be promising, showing that 91% of patients remained free of SVD after ≥5 years post-implantation (Arsalan and Walther, 2016). Currently, only the “NOTION” trial has reached a follow-up time of 10 years after transcatheter aortic valve implantation (CoreValve, Medtronic). However, the study reveals several limitations, making it difficult to draw definitive conclusions as reported before (Ternacle et al., 2024). After 10 years, the trial showed a mortality and re-operation rate of 65.5% and 4.3%, respectively. SVD occurred in 15.4% of patients, of whom 12.3% had a mean gradient ≥20 mmHg accompanied by an increase of their initial gradient by ≥10 mmHg (Thyregod et al., 2024). The surgical comparison group (bioprostheses) showed similar results. The relatively high mortality was attributed to the advanced age of the patients. In lower-risk patients, comparable studies have shown mortality rates of 10.0% (PARTNER 3) and 9.0% (Evolut Low Risk) after 5 and 4 years, respectively (Thyregod et al., 2024).

On the other hand, 10 years post aortic valve replacement utilizing the Ozaki procedure, the mortality and re-operation (due to SVD or endocarditis) rate was 24.8% and 8.8%, respectively. The mean gradient was 8.2 mmHg (Oz et al., 2023). A similar outcome was described in a systematic review analysing several studies assessing the Ozaki procedure (Badalyan et al., 2023).

Compared to prostheses from xenogenic material, the Ozaki procedure has shown favorable results regarding survival and hemodynamics, but has shown a higher reoperation rate. Although the data is not yet sufficient to make a statement about a potential improvement in durability, it is assumed that autologous NPT might exceed the lifespan of xenogenic prostheses (Prêtre and Sologashvili, 2018). Furthermore, the use of autologous NPT was described to be less infectious with excellent biocompatibility, and therefore a good choice for patients with endocarditis (Yepez and Ríos, 2021).

The application of the Ozaki procedure in pediatric patients is still relatively new. Therefore, follow-up periods are limited. However, a review of the existing studies shows very promising short-term results, primarily due to their excellent hemodynamic properties and early to mid-term durability, as well as the retention of annular growth potential (Wang et al., 2025).

The GrOwnValve procedure showed good valve functionality in a sheep model (Kiekenap et al., 2024) and is currently assessed in a First-in-Human trial in adult patients (Charité Universitätsmedizin Berlin, 2025). Although the GrOwnValve prosthesis may offer superior regeneration and somatic growth potential, due to its 3D-shaping and novel tissue treatment, definitive conclusions cannot yet be drawn. Initial clinical data are expected within the next year, and a study in pediatric patients is still pending.

However, the use of autologous pericardial tissue with regenerative potential could offer a significant improvement in heart valve treatment for adult but especially pediatric patients.

### Comparison of heart valve and pericardial tissue

To evaluate the adaptability of NPT for use as heart valve material, it is crucial to develop a deeper understanding of the native heart valve (NHV) structure.

**FIGURE 1:**
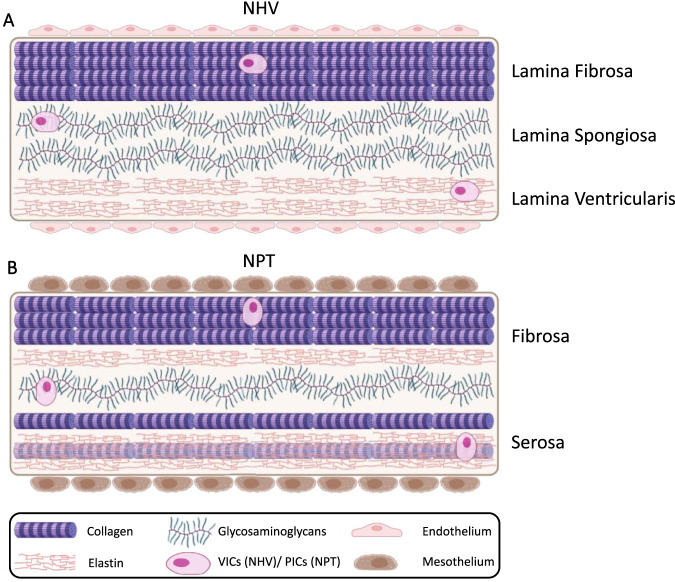
Simplified schematic representations of **(A)**: HVT (adapted from Rutkovskiy et al. (2017)) (Rutkovskiy et al., 2017), and **(B)**: NPT (based on the description of Rodriguez and Tan (2017)) (Rodriguez and Tan, 2017). Both tissues exhibit a similar composition of cellular and extracellular components. However, unlike NHV, NPT lacks the trilaminar architecture that confers anisotropic properties. In addition, NPT is covered by mesothelial cells, whereas NHV is covered by endothelial cells. Under mechanical stimuli in the new environment, the interstitial cells of NPT are expected to mimic the architecture of NHV over time. The figure does not account for fiber orientation. NHV, Native Heart Valve; NPT, Native Pericardial Tissue; VICs, Valve Interstitial Cells; PICs, Pericardial Interstitial Cells.

NHV consist of membranous leaflets, which are the most mobile parts of the valve (Heim et al., 2013). They possess a trilaminar architecture: lamina fibrosa, lamina spongiosa, and lamina ventricularis (Lamina atrialis for atrioventricular valves). The layers are reinforced by connective tissue and covered by endocardium (Misfeld and Sievers, 2007). All three layers are covered with resident valve interstitial cells (VICs) and the structural proteins collagen and elastin, which are intimately associated with the surrounding proteoglycans and glycosaminoglycans. These components form the extracellular matrix (ECM). However, the layers differ in their matrix composition: The lamina fibrosa (facing the outflow tract) is richer in collagen fibers, the lamina spongiosa (intermediate layer) is richer in proteoglycans, and the lamina ventricularis (facing the ventricle) in elastin (as shown in Figure 1A). However, all structural components are present in every layer (Rutkovskiy et al., 2017). Collagen fibers are primarily oriented in a circumferential direction, whereas elastin fibers exhibit a strong affinity for an alignment in a radial direction (Tesfamariam et al., 2019). Their varied distribution and alignment result in different mechanical properties across the layers (anisotropy), which is essential for a NHV to facilitate unidirectional blood flow (Kostyunin et al., 2020). This distinctive architecture enables nonlinear stress behavior, load damping, and high elasticity, allowing the valve to endure millions of cycles (Kostyunin et al., 2020; Zeeman, 1998).

Collagen, which is the most abundant protein in mammals, makes up the major component of the valve matrix. The term collagen encompasses a wide range of protein molecules that consist of three individual α-chains that are biosynthetically cross-linked and fold into a triple helix (Korossis, 2018). Depending on the tissue’s source, the composition of these collagen chains varies (Yepez and Ríos, 2021). Collagen accounts for 50% of the ECM of NHV, of which 74% is collagen I and 24% is collagen III(29). As the main load-carrying tissue element (Yepez and Ríos, 2021), it consists of strong, dense fibers that provide the NHV with the necessary tensile strength and resistance to extensibility (Brelje and Sorenson, 2009).

Elastin, on the other hand, is the main component of elastic fibers (Yepez and Ríos, 2021) and provides elasticity to the valve, which accounts for 14% of the ECM(29). It permits tissues to deform under load and recoil after their release (elastic recovery), which is crucial for NHV since they require undergoing repetitive stretch/relaxation cycles (Schornik et al., 2012; Yepez and Ríos, 2021).

Another structural component is glycosaminoglycans, which are composed of repeating disaccharides, including mainly hyaluronic acid, heparin/heparan sulfate, chondroitin sulfate/dermatan sulfate, and keratan sulfate (Casale and Crane, 2025). They are mostly linked to polypeptide chains forming proteoglycans (Yepez and Ríos, 2021). Glycosaminoglycans are important for energy absorption during the compression in the cardiac cycle (Straka et al., 2018). In addition, they contribute to cell hydration and structural scaffolding and reduce shear forces within the NHV by providing a lubricating property for the tissue due to its gel-forming character (Zeeman, 1998; Yepez and Ríos, 2021). They also play a key role in cell signaling, including the regulation of cell growth and proliferation, the promotion of cell adhesion, anticoagulation, adhesion, and wound healing (Zeeman, 1998).

There are two types of cells found in the NHV tissue: the endothelial cells, which cover the surface of the leaflets, and the interstitial cells, which form a network by communicating with each other and the ECM (Chester et al., 2014). During embryogenesis, a portion of the endothelial cells migrates into the leaflet matrix and undergoes endothelial to mesenchymal proliferation to become interstitial cells. Therefore, VICs have an endothelial origin (Rutkovskiy et al., 2017). This dynamic population exists in various phenotypes, including fibroblasts and alpha-smooth muscle actin (SMA) positive cells. The alpha-SMA cells encompass smooth muscle cells and an “activated” subset of fibroblasts, known as myofibroblasts, which replenish and remodel the valvular ECM and express contractile proteins (Schornik et al., 2012; Chester et al., 2014; Taylor et al., 2000). Therefore, the biological function of these cells is essential for maintaining the integrity of the leaflets and the overall function of a NHV, enabling lifelong maintenance and integrity. VICs are found in all cardiac valve layers but are most abundant in the lamina fibrosa (Chester et al., 2014). In the lamina fibrosa, where collagen I and III are denser, the cells are spindle-shaped and aligned along and connected to the fibers. In the lamina spongiosa and the lamina ventricularis, the VICs occur in a spread polygonal morphology (Straka et al., 2018). The varying cell phenotypes across the different layers can be attributed to the anisotropic forces within the tissue, which drive cell proliferation (Rutkovskiy et al., 2017). Depending on the flow and pressure patterns, the cells acquire specific properties and functions (Tesfamariam et al., 2019). It was observed that the phenotype of VICs is dependent on their mechanical stimulation, showing a higher stiffness in NHV from the left side (mitral and aortic valve) than from the right side (tricuspid and pulmonary valve) (Rutkovskiy et al., 2017), indicating the capability to adapt to the surrounding mechanical forces. VICs respond adaptively to their microenvironment, as dictated by the ECM or mechanical force applied to them, by releasing matrix components such as collagen, elastin, proteoglycans, and glycoproteins. Furthermore, they synthesize growth factors and biological mediators that trigger signaling cascades, which pathways can mediate both physiological and pathophysiological mechanisms. The synthesized matrix remodeling enzymes (metalloproteinases) can also regulate the phenotype of the cells, which in turn remodel the ECM (Chester et al., 2014).

The dynamic and complex cyclic mechanical stresses include (i) shear forces due to blood flow when the valve is open, (ii) bending stresses due to the cyclic opening and closing of the valve, and (iii) tensile stresses when the valve is closed (Alavi et al., 2013).

If there is a lack of living resident cells that can maintain valve homeostasis by repairing damaged structures, deterioration of a NHV over time is inevitable (Kostyunin et al., 2020). In conclusion, a NHV must be capable of adapting to repetitive changes in shape and dimension throughout the cardiac cycle and over a lifetime, which requires functional cellular and extracellular components (Yepez and Ríos, 2021).

NPT exhibits a cellular and ECM structure similar to that of NHV. Both pericardial and valvular tissues demonstrate comparable levels of hydroxyproline reflecting total collagen and elastin content, as well as similar mechanical properties (Filova et al., 2013), and overall tissue architecture (Tesfamariam et al., 2019). However, in contrast to the three-layer structure of the NHV, NPT shows a two-layered organization covered with mesothelial cells. These two layers, namely, the fibrosa and the serosa, resemble the lamina fibrosa and the lamina ventricularis of NHV, respectively. Collagen with interspersed elastic fibers is found in each layer, whereas the fibrosa consists of denser collagen bundles (Rodriguez and Tan, 2017). In the transition zone of the NPT, the collagen bundles are significantly less tightly packed, resembling the structure of the lamina spongiosa of heart valves. Glycosaminoglycans are distributed throughout the entire tissue (Straka et al., 2018) (as shown in Figure 1B). In the NPT, the collagen fibers are arranged in planes parallel to the membrane surface. The degree of preferential fiber orientation is variable. Elastin fibers run parallel to collagen fibers. The orientation is straight with extensive branching (Maurer et al., 2018).

Similar to VICs, the NPT contains pericardial interstitial cells (PICs), which encompass fibroblast and myofibroblast phenotypes and are capable of synthesizing ECM components. They exhibit a spread morphology in the serosal layer and a spindle-shaped morphology in the fibrosa, likely due to the higher collagen density in this layer. In both tissues, but less in the NPT, the interstitial cells mainly show a quiescent phenotype. In general NPT lacks a distinct organization, and the preferential fiber orientation is more variable compared to heart valve tissue. Furthermore, pericardial tissue shows higher collagen I, lower collagen III and elastin, and a similar glycosaminoglycan content compared to heart valve tissue (Straka et al., 2018).

### Remodeling potential due to mechanotransduction

The similarities of the tissues suggest that NPT provides a suitable tissue to replicate the capabilities of an NHV(5). However, for NPT to further adapt to NHV over time, to facilitate optimal blood flow, mechanosensitive PICs play a central role. This adaptation involves a transition from the initially less differentiated, two-layered structure of NPT (as shown in Figure 1B) to the differentiated, three-layered architecture of NHV (as shown in Figure 1A). In addition to mimicking NHV architecture, the adaptive remodeling potential of NPT is essential for maintaining prosthesis functionality, as factors such as improper fiber alignment can lead to various failure modes—including mechanical rupture and cyclic degradation—which in turn compromise durability (Alavi et al., 2013; Sellaro et al., 2007; Schoen and Levy, 2005). Furthermore, the stress-driven deterioration leads to damage of fibers, promoting the deposition of calcium (Kostyunin et al., 2020; Schoen and Levy, 2005). Consequently, rapid turnover of structural proteins is essential to withstand the repetitive deformation experienced during normal valve function (Korossis, 2018). Interstitial cells, therefore, actively respond to their local cellular and extracellular matrix environments, as well as their hemodynamic environment, via mechanotransduction. This adaptability can even occur independently in isolated parts of the NHV, a process referred to as dynamism (Chester et al., 2014).

Mechanotransduction can be classified into two types: centralized and decentralized. Centralized mechanotransduction involves the direct interaction of mechanical forces with a surface receptor and its transmission to downstream signaling cascades. In this process, mechanical forces are transmitted from the ECM via the mechanosensitive transmembrane proteins, which serve as heterodimeric adhesion receptors, into the cell.

**FIGURE 2:**
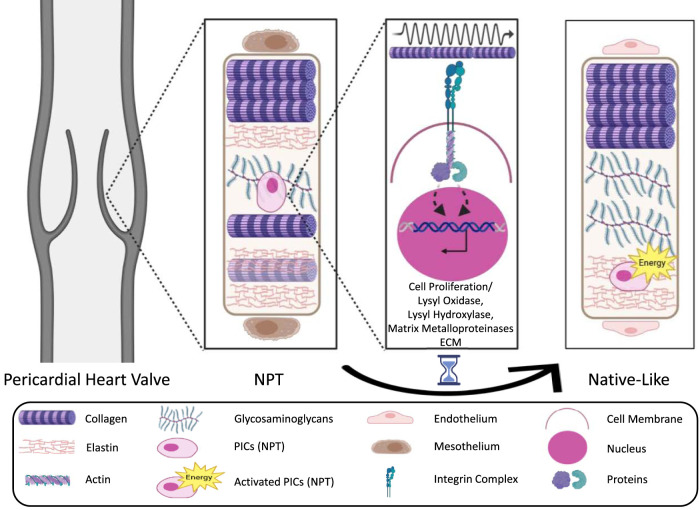
Simplified schematic representations of the ECM–integrin–cytoskeleton–nucleus pathway as an example of mechanotransduction (adapted from Tietze et al. (2020)) (Tietze et al., 2020). The illustration depicts a pericardial heart valve adapting toward a native-like heart valve structure in response to mechanical forces, which drive the differentiation of quiescent interstitial cells into an activated phenotype capable of ECM production, remodeling, and cross-linking through enzyme secretion. ECM, Extra Cellular Matrix; NPT, Native Pericardial Tissue; VICs, Valve Interstitial Cells; PICs, Pericardial Interstitial Cells.

One example is the ECM–integrin–cytoskeleton–nucleus pathway: In response to mechanical stimuli from the environment, integrin-based focal protein complexes assemble at the cell membrane and bind to ECM complexes like collagen or fibronectin. These complexes transmit the mechanical signals to the cytoskeleton. The proteins (especially actin) within the cytoskeleton, in turn, activate mechanosensitive transport proteins (e.g., paxillin, zyxin, yes-associated protein), which translocate the signals via “linker of nucleoskeleton and cytoskeleton” proteins into the nucleus. Depending on the external mechanical stimuli, they bind to transcription factors triggering a signaling cascade, resulting in the up- or down-regulation of various transcripts and gene expressions, and subsequently to the proliferation or differentiation of the cell or remodeling of the ECM(7,16). Therefore, quiescent interstitial cells could proliferate into an activated type capable of producing, remodeling, and cross-linking ECM through the production of several enzymes (Straka et al., 2018) (as shown in Figure 2).

In contrast, decentralized mechanotransduction is not characterized by a central signaling pathway, but rather by a localized deformation of the cytoplasm by mechanical stimuli. Intracellular components are in equilibrium between tension and contraction. The heterogeneous distribution of components generates local maxima and minima of mechanical stresses, which cause signal transduction to the nucleus and thus also a proliferation or differentiation of the cell or remodeling of the ECM, respectively (Taylor et al., 2000).

Straka et al. (2017) cultured a three-leaflet heart valve construct of living human NPT in dynamic conditions up to 4 weeks. They observed a threefold increase in PICs and a similar histological structure compared to NHV, indicating that PICs proliferate into an activated VIC-like phenotype under dynamic mechanical conditions. The conditioned NPT showed a comparable secant elastic modulus to NHV tissue. Furthermore, they showed that pericardial heart valve constructs possess optimal hemodynamic properties, similar to NHV. Later, the research group confirmed their results, showing that the human NPT consists of PICs with similar properties to those of human VICs, being capable of responding to mechanical stresses and synthesizing new ECM, including collagen I, elastin, and glycosaminoglycans (Straka et al., 2017). VICs (and PICs) are capable of reacting with a compensatory adaptive response to the body’s changing hydrodynamic and biochemical parameters (Kostyunin et al., 2020).

## Discussion

A living tissue that preserves its biological sensing mechanisms and can adapt its structureand function to the surrounding environment is essential for effective heart valve therapy. Mechanosensitive cells are key to enabling the tissue to remodel into a native-like structure, allowing it to withstand hemodynamic forces over extended periods (Taylor et al., 2000). NPT presents a promising option in this context, as it shares structural similarities with NHV and possesses all the necessary prerequisites for environmental adaptation.

The main technical challenge in developing a heart valve from autologous NPT with remodeling potential is to protect it from enzymatic degradation while simultaneously preserving tissue viability. A common strategy to counteract degradation is chemical cross-linking of the tissue’s structural proteins (Zeeman, 1998), (Balguid et al., 2007). Already in 1969, it was shown that insufficiently cross-linked autologous tissue is highly susceptible to enzymatic breakdown (Carpentier et al., 1969). Today, the most widely used cross-linking agent is glutaraldehyde (Sheehy et al., 2018). However, while glutaraldehyde effectively stabilizes the ECM, its cytotoxicity results in the implantation of non-viable tissue ((Bezuidenhout et al., 2009), (Prêtre and Sologashvili, 2018)). This creates an inherent trade-off: cross-linking prevents enzymatic degradation but eliminates cell viability, whereas omitting treatment preserves cell viability but leaves the tissue vulnerable to denaturation. Furthermore, it was reported that the durability of a heart valve prosthesis without living cells depends on the stability of the cross-links within the ECM(13). Given that enzymatic degradation poses the more immediate risk, current clinical practice still relies on glutaraldehyde treatment as the standard approach. To solve this problem, various post-glutaraldehyde-detoxification methods, like amino acids, glycine, heparin, hyaluronic acid (Remi et al., 2011), or coating methods, like methacrylated chondroitin sulfate hydrogel (Lopez-Moya et al., 2018), have been investigated. The major heart valve prosthesis manufacturers Edwards and Medtronic also use detoxification methods after glutaraldehyde treatment, namely, RESILIA tissue technology (Edwards, 2024) and AOA anti-calcification treatment (Medtronic, 2025), respectively. Furthermore, besides chemical cross-linking agents, biological and physical ones have been investigated (Delgado et al., 2015). One physically cross-linked and commercially available product is the PhotoFix bovine patch from Artivion (2025).

Conditioning the tissue before implantation may offer an alternative to using cross-linking agents, allowing the formation of natural, non-cytotoxic cross-links (Straka et al., 2017). Therefore, the collagen’s lysine residues are partly hydroxylated to hydroxylysine and subsequently oxidized by lysyl oxidase to allysine. The aldehyde groups of allysine then form natural cross-links with the amino groups of neighboring lysine or hydroxylysine residues (Zeeman, 1998). Maintaining cell-cell and cell-matrix adhesion receptors is particularly crucial in this regard (Straka et al., 2018). However, this approach involves a high level of regulatory effort and does not offer guaranteed protection against enzymatic degradation after re-implantation.

To date, only chemically cross-linked tissues have been considered suitable for clinical use in heart valve therapy. This suggests that the most promising strategy lies in optimizing tissue treatment protocols (Trimaille et al., 2025), rather than abandoning it. For instance, the Ozaki method demonstrates that a significantly shortened glutaraldehyde treatment (10 min instead of 24 h) allows a fraction of cells to survive the process (Gardin et al., 2021). Gentle stabilization of the ECM can preserve PIC viability, enabling them to respond to mechanical stresses through adaptive remodeling and therefore to mimic the native fiber structure of NHV (Straka et al., 2017).

However, long-term clinical data are crucial to assess the remodeling potential of pericardial prostheses and their ability to transform into native-like heart valves.

## Abbreviations

ECM, Extracellular Matrix; PICs, Pericardial Interstitial Cells; NHV, Native Heart Valve; NPT, Native Pericardial Tissue; SMA, Smooth Muscle Actin; SVD, Structural Valve Degeneration; VICs, Valve Interstitial Cells.
